# A Presentation of Central Communication Aspects in the Patient–Provider Relationship—Potential Learnings for Community Pharmacists

**DOI:** 10.3390/pharmacy8040241

**Published:** 2020-12-18

**Authors:** Nima Jowkar, Nina Fjeldsø, Lotte Stig Nørgaard, Sofia Kälvemark Sporrong, Ramune Jacobsen, Susanne Kaae

**Affiliations:** 1 Social and Clinical Pharmacy, Department of Pharmacy, University of Copenhagen, 1165 Copenhagen, Denmark; Nima1231@yahoo.com (N.J.); ninaf1991@hotmail.com (N.F.); lotte.norgaard@sund.ku.dk (L.S.N.); or kalvemark-sporrong@farmaci.uu.se (S.K.S.); ramune.jacobsen@sund.ku.dk (R.J.); 2 Department of Pharmacy, Uppsala University, 752 36 Uppsala, Sweden

**Keywords:** pharmacy, communication, relationship

## Abstract

Several studies have shown that communication between patients and HCPs is still not optimal in integrating patients’ perspectives on how best to manage their diseases and their medication. One such area where encounters between patients and professionals still needs to develop to better incorporate the patient’s perspective is pharmacy practice. The aim of this study was therefore to explore and present new typologies and communication aspects of HCP-patient relationships since a comprehensive literature search in 1997 conducted by Sondell and Söderfeldt, and relate the findings to pharmacy practice. In total, 11 articles were identified by applying the techniques of a purposeful literature search. The articles covered aspects of: shifting and adapting roles during the encounter, techniques to ensure individualizing in the encounter, avoiding inappropriate routines, coping with internet-informed patients, achieving mutual goals, dealing with uncertainty including avoiding rigid preconceptions, using social conversation, incorporating patients’ prior experiences, aligning language, adapting greetings and exploring the cultures and communication patterns of patients from other ethnic backgrounds. The variety of these communication aspects points to the immense complexity of communication as a practice discipline. This article has presented some of the literature that pharmacists can consult, in the endeavor of improving their communication practices.

## 1. Introduction

Communication between patients and health care professionals (HCPs) plays an essential role in ensuring optimal care, including the management and prevention of medication errors. Communication is a complex process involving, among other things, components of power and roles. Communication is shaped by what we bring into the encounters based both on our expectancy set formed by personal, collegial, and cultural experiences and practices [1], and by our individual communication style, i.e., the way in which we as individuals interact and exchange information with others [2].

The communication culture in health care has developed over time and is continuously evolving. At the end of the 19th century, physicians were at the “top” of the healthcare hierarchy and patients were to obey the physician’s directions [3,4,5]. In the second half of the 20th century, public discourses concerning humanity, equality, and relationships appeared, requesting HCPs to treat patients as equal partners. Additionally, patients began to demand more information and involvement in their treatments [3,5], thus replacing a “silent” patient with a more “critical” one [4]. Despite these developments, several studies have shown that communication between patients and HCPs is still not optimal when it comes to integrating patients’ perspectives on how best to manage their diseases [6,7,8]. One area in which encounters between patients and professionals still needs to develop to better incorporate the patient’s perspective is community pharmacy practice [9,10].

To advance communication in community pharmacy practice, a thorough understanding of what occurs in medical encounters between a patient and a HCP, including what to be aware of when communicating, is highly needed. A comprehensive study about communication between HCPs and patients was a literature review published back in 1997 by Sondell and Söderfeldt, who aimed to describe different types of doctor/dentist–patient relationships, and the external factors influencing the relationships to further communication in dental care [11]. The authors identified two types of models in the literature: the normative (arguing that some types of encounters are more appropriate than others for example the bio-psychosocial model being more acceptable than the biomedical) and the empirical (describing what actually happens). Furthermore, they identified four types of relationships (with a difference as to how patients are involved in the encounter), and 16 different communication models describing a variety of factors involved in HCP–patient encounters [11]. A lot of useful information about various aspects of HCP–patient communication, also with regard to community pharmacy practice, can be derived from the review, such as: who controls what happens during a health care encounter, what aspects should be considered in order to create more equal relationships, and what are the relevant patient outcomes? The authors defined ‘relationships’ or ‘interactions’ as ‘the situation in which humans act on each other. An act towards someone is followed by an act/answer of any kind from someone receiving’. A central point in the work by Sondell and Söderfeldt was thus the implicit duality in all encounters between patients and providers, and the mutual responsibility held by both to make the communication “work”.

The need to focus on the dynamic interaction between the two parties when studying communication between patients and HCPs, rather than aspects pertaining to just one of the parties, has also been stressed by researchers in social pharmacy. In 2006, Shah and Chewning published a review of studies in the US exploring communication between patients and community pharmacists [12]. The main objective of this review was to summarize how researchers have conceptualized and measured pharmacist–patient communication in order to suggest how to evaluate pharmaceutical care services in the future. The authors found that existing studies focused primarily on the pharmacist’s information providing behavior, including the extent and content of the provided information, and the manner in which the pharmacist brought forward the information. The literature therefore seemed to overlook how patients influence the interaction between pharmacists and patient. Based on the included studies, Shah and Chewning provided a number of suggestions for how to conduct research in the future in terms of stances and focus points, data collection methods, analysis techniques and relevant theoretical underpinnings in order to better capture the dyadic interaction between the two parties, including gaining a more in-depth understanding of how and why it develops.

On a more practical level, Ilardo and Speciale (2020) published a narrative review discussing how the pharmacy profession could develop further by improving their communicative skills with patients, in particular on how to best communicate with patients with varying degrees of health literacy [13]. Ilardo and Speciale thereby also stressed the need for pharmacists to become truly patient centered. Several communication models were presented by the authors including: the patient-centered communication dimensions by Mead and Bower and the Alberta (Canada) Comprehensive Annual Care Plans. Furthermore, 11 general areas of key communication skills for pharmacists in conversations with patients were listed, including the aspects of: opening, building rapport, questioning and suggesting/advising. Finally, communication strategies developed by Kripalani and Jacobson (such as simplifying one’s language and techniques supporting patients’ understanding and recalling of advice) were given. This review thereby illustrated several communication models with a range of dimensions or sub-categories to take into account by pharmacists. The strategies by Kripalani and Jacobson provided some specific input into how pharmacists should incorporate patients’ perspectives, but apart from that, the presented models did not always specify exactly how communication could be enacted.

Continuing from Ilardo and Speciale (arguing that the communication should be patient centered) and taking into account the points by Sondell and Söderfeldt and Shah and Chewning (that the dynamic interaction between the patient and the provider should be the focus point when communicating in health care), we aimed at presenting and discussing ‘new’ communication aspects of the HCP–patient relationship. Hence, the focus of this paper is to discuss more recent communication aspects that pertain to particular elements in the interaction between the two parties (patient and HCP), rather than just one of the parties. At the same time, we want to present aspects which might be consulted by community pharmacists in order to incorporate them into their daily practice. The paper thus has a practice agenda rather than a research-based agenda as compared to the review by Shah and Chewning, and is more in line with the original aim of Sondell and Söderfeldt, though focusing on another group of HCPs, here community pharmacists.

## 2. New Communication Aspects in the Patient–Provider Interaction of Relevance for Pharmacists

In the following, we will introduce the pharmacist reader for a variety of communication aspects which are all of relevance, we trust, for the interaction between a pharmacist and a patient. The focus has thus been on materials that illustrate different approaches on how to better engage with patients, by making the communication more individual and personally relevant for the patient. Despite the fact that professions vary in their role perception [14], and that communication in the pharmacy differs from other types of meetings due to the pharmacy staff’s limited knowledge about their patients’ clinical issues, lack of privacy in the pharmacy and that medicinal products are purchased during the encounter, pharmacy practice can presumably still learn from other areas of health care practice. Therefore, we also discuss this type of literature. In total, eight different communication aspects will be covered, dealing with:

- shifting and adapting roles during the encounter,
- coping with Internet-informed patients,
- achieving mutual goals,
- dealing with uncertainty,
- adopting social components,
- aligning language,
- building cultural competency,
- building an equal relationship.

We will present the communication aspects one by one, followed by suggestions for how each one illustrates new insights as compared to the review by Sondell and Söderfeldt, since this review was also focused on the interaction between HCP and patients. We then discuss the specific relevance of the aspects for community pharmacy communication.

### 2.1. Shifting and Adapting Roles During the Encounter

A new approach to describing pharmacist–patient relationships was undertaken by Austin et al. in 2006, who identified the following five different role pairs: analytic–authoritative, emotive–interactive, opportunistic–expedient, reliant–paternalistic, and autonomous–informative [15]. Austin et al. based the foundation of the role pairs on the type of ‘patient’ and the response of the pharmacist accordingly. Hence, if, for example, the patient took a more ‘consumer-oriented’ role in the pharmacy encounter, displaying a high need for personal control, the pharmacist adopted his/her role in this case to provide evidence and facts, leaving it up to the patient how to use the information.

This approach seems to expand on how power relationships develop as described by Sondell and Söderfeldt, where the roles seemed to stem from the HCP’s initial choice of relationship. Austin et al., on contrary see the pharmacist adopting their behavior towards the patient. Who initiates the orientation of the relationship in a medical encounter is an important aspect to consider since it may affect the expectations, roles, and responsibilities, which could influence the encounter. With regard to furthering pharmacy practice, the study underlines some possibilities in the role-taking and initiation of a pharmacy encounter, including shifting between roles.

### 2.2. Coping with Internet-Informed Patients

In a study by Caiata-Zufferey et al. (2012), physicians’ communication strategies for encounters with Internet-informed patients were investigated [16]. The Internet can result in the patient–physician interaction becoming more complex, as interaction patterns and expectations change. A typology of four communicative strategies used by physicians was therefore identified either as physician-centered or patient-centered: (i) “Resistance to online information:” physicians invalidated and discounted the patient’s achieved knowledge from the Internet, (ii) “Repairing online information:” physicians warned about Internet searches, (iii) “Co-construction around online information:” physicians showed understanding of the patient’s point of view by assessing the online information brought in his/her attention at the consultation, and (iv) “Enhancement of online information:” physicians empowered the patient by encouraging him/her to take active and critical measures in their Internet research.

The typology shows a variety of new techniques that can be employed by the HCPs, since the study by Sondell and Sönderfeldt (1997) did not include technological aspects such as the Internet, which is an integral part of the HCP–patient relationship today. ‘Enhancement of online information’ can be seen as a variety of the consumerist relationship also described by Sondell and Söderfeldt, where the physician meets the patient’s expectations for information and tries to empower the patient. Though consumerism in the pharmacy is not a new concept, discussion of how it applies to patients’ use of new technologies including the Internet is a relatively new dimension to pharmacy practice. Every day, pharmacists meet patients who have consulted the Internet before visiting the pharmacy and hence, pitfalls in the communication or empowering techniques can be derived from this study.

### 2.3. Achieving Mutual Goals

A systematic review by Sabater-Galindo et al. (2016) examined theoretical interaction models between HCPs and patient in terms of relevance for community pharmacy [17]. Eight models were identified and classified into two approaches: (i) models describing the HCP–patient relationship as an interactive care process (Peplau’s Interpersonal Relations model, The Intersystem Patient Care Model, The Generic Model of Psychotherapy and Information-exchange Model of Medical Consultation), and (ii) models that describe the nature of the HCP–patient interaction and its association with the efficacy of health care (Theory of the Nursing Process Discipline, the Theory of Goal Attainment, the Model of the Interaction Phase of Symptom Management in a Client–nurse Relationship and the Three-Dimensional Puzzle Model of Culturally Congruent Care). In addition, factors influencing the professional–patient relationship were characterized for each model as (i) modifiable factors (knowledge, needs, values, expectations, beliefs and perceptions), and (ii) non-modifiable factors, identified in patients only (previous healthcare experiences and encounters, sociodemographic factors, including age, gender and socioeconomic status, and cultural diversity).

There was no overlap between the described models and the models presented by Sondell and Söderfeldt (1997) except for Frederikson’s model of ‘Information-exchange model of medical consultation. The authors themselves highlight one of the models found, ‘The Theory of Goal Attainment’ by King, which explains how the perceptions of patients and HCPs regarding objects, persons, and events influence their social behaviors and thereby social interaction in medical encounters. ‘The Theory of Goal Attainment’ thereby provides a basis for pharmacists to explore and understand how they can better achieve mutual goal-setting by tapping into the basis of patients’ perceptions, perceptions the pharmacist can then attend to in order to obtain the desired health goals of the individual.

### 2.4. Dealing with Uncertainty

Bylund et al. (2012) gave a brief overview of selected interpersonal theories and models, and presented examples of their use in healthcare communication research (Goals–Plans–Action Theory, Uncertainty theories, Action Assembly Theory, Communication Accommodation Theory, Facework and Politeness Theory, Speech Codes Theory, Social Penetration Theory, Communication Privacy Theory and Management Theory) [18]. The uncertainty theories were divided into two categories: the Uncertainty Reduction Theory and the Uncertainty Management Theory. The assumption behind classic uncertainty theory is that an individual’s primary goal in communication is to increase predictability and decrease uncertainty of one’s own behaviors and the behaviors of others, by (a) striving to predict the communication behaviors before an interaction, or (b) retroactively seeking to explain behavior. Uncertainty is thus a fundamental human condition that can stir a wider range of emotions and anxiety also in communication in health care; although, newer uncertainty theories question if people are indeed always motivated to reduce uncertainty.

The uncertainty theories thereby add a new existentialistic aspect to communication compared to the communication models and factors described by Sondell and Söderfeldt. In terms of pharmacy practice, the theories explain how both pharmacists and patients might sometimes, in their efforts to reduce basic uncertainty, jump to conclusions about the other party too soon, and thus misinterpret their motives. The theories point to the need for becoming more consciously aware of this fundamental psychological need when communicating and instead make effort to check if our interpretations are correct.

### 2.5. Using Social Conversation

Greenhill et al. (2011) explored communication between pharmacists and patients through the application of the comprehensive Calgary-Cambridge Guide (CCG) developed for appointment-based pharmacist–patient consultations [19]. The CCG comprises no less than 71 communication skills, such as how to initiate a dialogue, gather information, provide structure, build relationships, explain/set up plans and close the dialogue. Though comprehensive, Greenhill et al. still identified one additional skill not mentioned in the guide, namely social conversation/casual conversation about non-medical issues (such as social matters, sport, etc.). Greenhill et al. (2011) thus suggested the incorporation of social conversation as a relationship-building skill in the CCG.

The importance of social conversation as part of a medical encounter serves other purposes than the HCP simply using it to explore patients’ backgrounds (as can be done during the bio-psychosocial relationship), such as to build a more equal and trusting relationship. Adapting a list with 71 communication skills into a pharmacy encounter lasting three minutes is of course not possible. The part of social or causal talk is certainly already practiced today in community pharmacy; however, considering exactly when and how to facilitate non-medical talk as part of the interaction over a pharmacy counter to better liaise with patients to further relevant talk about the medicines could be an important aspect to further patient-centered counselling.

### 2.6. Aligning Language

In the US, Vrana et al. (2018) studied the degree of semantic similarities in language used in communication between a patient and a physician during medical encounters, i.e., how similar or coherent the language is, which was assumed to influence the interaction [20]. The study described how the similarity of communication language depended on gender and ethnicity, with white physicians’ conversations exhibiting lower semantic similarities with their patients than physicians with an Indian/Pakistani background. Likewise, female patients exhibited greater communication similarities with their physicians than male patients [20].

Focusing on how language is used in the dialogue provides an opportunity for pharmacists, as this study suggests that the development and outcome of an encounter can be influenced and changed by aligning the language across age, gender, ethnicity, etc. Ilardo and Speciale discussed simplifying language, but aligning language on a semantic level, considering the possible differences in the underlying meanings inhered in the language from various positions, hereby firstly acknowledging that multiple interpretations are possible, is a topic not often discussed in the pharmacy setting. This aspect is however crucial for how the encounter develops.

### 2.7. Building Cultural Competency

To build on aligning understandings, in 2009, Teal et al. suggested a ‘culturally competent communication model’ [21]. The model explores HCPs’ communication and cultural sensitivity competences, suggesting that the HCPs, while practicing, should not only improve communication skills but also learn about and become culturally competent. Thus, HCPs should get acquainted with how possible differences in words and non-verbal expressions may be understood, in order to be ready to adapt their communication.

Many pharmacists meet patients with different cultural backgrounds daily. Being more curious towards and investigating these patients’ understanding of medicines and the provided counselling, especially those specific ethnic groups often visiting the pharmacy, is a good investment to further patient centered communication.

### 2.8. Building an Equal Relationship

Barrere et al. (2007) investigated nurse–patient communication with regard to what specific actions during their encounters contribute to making the relationship more or less equal [22]. The choices made by nurses on how to interact with patients included: how nurses present themselves to (new) patients and by which name they address the patient; whether nurses focused more on personal or medical aspects during their talks with patients (and when and why to shift between personal and professional subjects), and if cues from patients were recognized and addressed.

The study by Barrere et al. thereby refers to the shift between private and professional roles and might appropriately be used for understanding which specific communication strategies to use to turn an encounter into a more patient centered and individual one. As presented also by Greenhill et al. [19], the importance of social/personal talk as a part of the medical encounter was highlighted along with how to best address the patient [20]. The study by Barrere et al. thus provides concrete and relevant communication strategies for pharmacists to consider on how to make encounters more equal and personal.

## 3. Conclusions

A variety of health care communication models, stances, aspects and results, all of relevance for the interaction between community pharmacists and patients, have been presented. The difference in the communication aspects points to the immense complexity of communication as a practice discipline. Rather than being overwhelmed and discouraged by this fact, we should rather perceive it as an opportunity for pharmacists. By taking small steps incorporating one new relevant communication aspect at the time, according to your individual background and the circumstances in which you practice as a pharmacist, could be a way to further patient counselling. This article has presented some of the literature that pharmacists can consult with to support them in this endeavor.
